# Associations between potential inflammatory properties of the diet and frequency, duration, and severity of migraine headaches: a cross-sectional study

**DOI:** 10.1038/s41598-022-06819-y

**Published:** 2022-02-21

**Authors:** Seyed Mojtaba Ghoreishy, Gholamreza Askari, Hamed Mohammadi, Marilyn S. Campbell, Fariborz Khorvash, Arman Arab

**Affiliations:** 1 Department of Clinical Nutrition, School of Nutritional Sciences and Dietetics, Tehran University of Medical Sciences, Tehran, Iran; 2 Department of Community Nutrition, School of Nutrition and Food Science, Food Security Research Center, Isfahan University of Medical Sciences, Isfahan, Iran; 3 Department of Kinesiology and Health Promotion, University of Kentucky, Lexington, KY USA; 4 Isfahan Neurosciences Research Center, Alzahra Hospital, Isfahan University of Medical Sciences, Isfahan, Iran

**Keywords:** Migraine, Health care

## Abstract

Despite a large body of literature on the association between the dietary inflammatory index (DII) and various chronic diseases, limited knowledge is available regarding the association between DII and migraine. Therefore, we assessed the relationship between the DII and migraine characteristics, including duration, frequency, and severity of migraine headaches, Headache Impact Test-6 (HIT-6), and serum levels of nitric oxide (NO). This population-based cross-sectional study was conducted from August 2019 to June 2020 among 262 patients (38 men and 224 women; 20–50 years). A 168-item semiquantitative food frequency questionnaire (FFQ) was gathered to evaluate dietary intake, and subsequently, an energy-adjusted DII score was calculated. After controlling for potential confounders, an increase of 3.48 in headache frequency was observed when the DII score increased from − 4.04 to − 1.83 (β = 3.48; 95% CI 1.43, 5.54). In the crude model, headache duration tended to be inversely associated with DII in the subjects with the pro-inflammatory diet compared to those with the anti-inflammatory diet (β = − 0.22; 95% CI − 0.46, 0.02). After adjustment for confounders, those with the highest DII values were at a higher risk of severe headaches than those with the lowest values (OR = 2.25; 95% CI 1.17, 4.32). No other significant results were found in terms of the association between DII and HIT-6 or serum NO levels. We found evidence suggesting that higher adherence to a diet with anti-inflammatory properties was significantly and inversely related to headache frequency. Furthermore, our results suggest that the DII score is substantially related to migraine severity.

## Introduction

Migraine is a chronic neurological disorder defined by unilateral repeated episodes of headaches, sometimes accompanied by nausea, vomiting, phonophobia, and photophobia^1^. Functional disorders following headaches are seen in 90% of patients, which can eventually affect various aspects of daily life such as work, social, and family relationships^2–5^. Furthermore, migraine has many indirect and direct costs for patients and society^6–8^. According to the World Health Organization (WHO) report, migraine is the seventh most debilitating disease worldwide^9,10^, and it is more commonly seen among women. In fact, migraines are two to three times more common in women than men, with a global prevalence of 14.7% for both sexes^11^.

Migraine pathology is not fully understood; however, previous research has indicated that the etiology of migraine could be composed of both environmental and genetic factors^12^. Migraine attacks can be prompted by vascular inflammation, corticotropin-releasing hormone, neurogenic and trigeminovascular system activation, degranulation of mast cells located in the dura, and nitric oxide (NO)-like trinitroglycerin^13–15^. Previous work has shown that NO has an important role in trigeminovascular inflammation occurring during migraine attacks^16^. Earlier studies have illustrated that a migraine headache is significantly related to systemic inflammation and oxidative stress^13,17–19^.

Dietary factors have a significant role in the modulation of chronic inflammation^20^. Studies have shown the relationship between specific eating patterns and neurological disorders; notably, the intake of foods with anti-inflammatory properties (e.g., beans, fruits, and vegetables) is correlated with a lower risk of Alzheimer’s disease^21^, Parkinson’s disease^22^, and multiple sclerosis^23^. In contrast, a Western dietary pattern^24^, full of nutrients or foods with pro-inflammatory effects, has been correlated with a higher risk of depression^25^.

The Dietary Inflammatory Index (DII) has been a useful tool used in nutritional research to evaluate inflammatory potential by considering pro-inflammatory and anti-inflammatory attributes of special foods or dietary compounds, such as macronutrients, vitamins, minerals, and flavonoids^26,27^. Although previous studies indicated that inflammation and oxidative stress triggered a migraine headache^28^, the association between DII and migraine was not assessed in these studies. One previous cross-sectional study including 266 women has shown that there was an association between headache frequency and DII. However, no relation was observed between headache duration or migraine severity and the DII score after adjusting for traditional risk factors^29^. Therefore, the current study was designed to further explore associations between migraines and DII using additional indices including the Headache Impact Test-6 (HIT-6) and NO. Given the limited information and the small number of studies considering the association between DII and migraine, the present cross-sectional study aimed to assess the association between DII and migraine characteristics including duration, frequency, and severity of headache as well as HIT-6 and serum levels of NO in an Iranian population diagnosed with migraine.

## Materials and methods

### Participants

**Figure 1:**
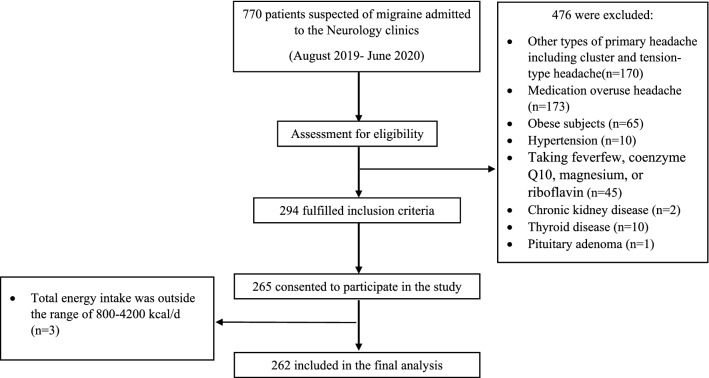
Flow chart of the participants selection process.

The present cross-sectional study was conducted from August 2019 to June 2020 in Isfahan, Iran. The study population was migraine patients who were admitted to the Imam Musa Sadr and Khorshid neurology clinics both affiliated with Isfahan University of Medical Sciences. Upon predefined inclusion and exclusion criteria and using a simple random sampling method, a total of 262 patients (38 men and 224 women; 20–50 years) were eligible to be enrolled in the current study. Prior to the enrollment, patients were informed regarding the aims, study procedures, and potential outcomes and their participation was voluntary (Fig. 1). Included patients suffer from migraines as defined by expert neurologic diagnosis using the International Classification of Headache Disorders (ICHD–3) criteria^30^. Patients were included in the study if they met the following inclusion criteria: (1) a Body Mass Index (BMI) between 18.5 and 30.0 kg/m^2^ and (2) diagnosis of migraines by a neurologist (F.K) based on the aforementioned criteria. Exclusion criteria included: (1) patients with a history of hypertension, diabetes, thyroid disease, cardiovascular disease, renal disease, cancer, hepatic disease, or other neurological disorders; (2) use of herbal and nutritional supplements including feverfew, coenzyme Q10, magnesium, or riboflavin; or (3) daily energy consumption outside the range of 800–4200 kcal/day (3347–17,573 kJ/day)^31^. The study protocol was approved by the Isfahan University of Medical Sciences Research Ethics Committee (IR.MUI.RESEARCH.REC.1398.352). Written informed consent forms were received from all participants. All study protocols were conducted according to the Declaration of Helsinki, and results were reported based on the strengthening the reporting of observational studies in epidemiology (STROBE) statement for cross-sectional studies.

### Dietary intake assessment

A 168-item semi-quantitative Food Frequency Questionnaire (FFQ), a Willett-format questionnaire modified for Iranian foods, was used to evaluate the study participants' usual dietary consumption; the validity and reliability of this questionnaire have been established in a prior study^32^. All questionnaires were administered by a skilled dietitian. The FFQ contained a list of foods with a standard serving size for each. On a daily, weekly, or monthly basis, participants were requested to report their frequency of consumption of each food item over the previous year. Using home measurements, the portion sizes of consumed foods were estimated in grams^33^. The reported frequencies of intake of the various foods and beverages were then coded and analyzed for daily energy and nutrient content using Nutritionist 4 software (First Databank, Hearst Corp., San Bruno, CA, USA), which has been modified for Iranian foods.

### Calculation of DII

The method previously reported by Shivappa et al. for calculating DII was used in the current analysis^26^. Out of 45 food items proposed by Shivappa et al., the DII score was calculated using 32 dietary parameters including energy; carbohydrate; protein; fat; cholesterol; fiber; monounsaturated fatty acids (MUFAs); polyunsaturated fatty acids (PUFAs); saturated fatty acids (SFAs); trans fatty acids (TFA); omega-3 fatty acids; omega-6 fatty acids; vitamin B12; folic acid; pyridoxine (B6); niacin (B3); riboflavin (B2); thiamin (B1); vitamins A, C, D, and E; beta carotene; magnesium; zinc; selenium; iron; caffeine; pepper; garlic; onion; and green/black tea. The energy-adjusted amounts of all nutrients were calculated via the residual technique^34^. Then, to get the z-score, the “standard global mean” was subtracted from the quantity of food and divided by the “global standard deviation” (SD). Standard global means and SDs for each dietary item were obtained from the article by Shivappa et al.^26^. To decrease skewness, this value was converted to a centered percentile score and was multiplied by the effect score for each of the food items using the numbers reported by Shivappa et al.^26^. Finally, we added the DII scores from all dietary components of individual participants to get the total energy-adjusted DII score for each subject. Positive values (higher DII scores) indicate a diet with pro-inflammatory properties, whereas negative values (lower DII scores) indicate a diet with anti-inflammatory properties.

### Assessment of migraine

The HIT-6 tool, a 6-item validated questionnaire^35^, was employed to determine if migraine affects the quality of life of participants. This questionnaire contains 6 questions with 5 options for each ranging from never (scored as 6) to always (scored as 13) with a total score of 36–78. The overall score was categorized as little or no impact (≤ 49), some impact (50–55), substantial impact (56–59), and severe impact (≥ 60)^36^.

A 30-day headache diary was given to all participants with written and verbal instructions for completion. Participants were required to record clinical features of migraine, including headache severity, duration, and frequency for a month. The severity of headaches was evaluated using the visual analog scale (VAS) questionnaire (ranged from 0 to 10, with “0” meaning no pain and “10” the worst imaginable pain) and the overall score was categorized as mild (1–3), moderate (4–7), and severe (8–10) headache pain as suggested by previous studies^37^. The number of attacks per month (frequency) and mean duration of headache attacks per month (duration) were also examined.

NO was measured via the Griess method using a serum sample of patients which was taken after 8 h of fasting (Kiazist Life Sciences, Iran). Blood sampling was done the next day after the completion of the questionnaires.

### Assessment of other variables

Demographic characteristics including sex, age, smoking status, marital status, number of family members, and medications were obtained using a questionnaire. Participants reported their level of physical activity using a validated version of the International Physical Activity Questionnaire (IPAQ) for 7 days^38^. A mercury sphygmomanometer (Riester, Germany) was used to measure blood pressure. Weight was measured in minimal clothing with a digital scale (Omron BF511, Omron Corp., Kyoto, Japan) to the nearest 100 g. Height was measured in a standing position without shoes by a wall tape meter to the nearest 1 mm. The BMI was calculated using weight (kg) divided by squared height (m^2^).

### Statistical analysis

Mean ± standard error (SE) and number (percent) were used to express the quantitative and qualitative variables, respectively. The tertiles of the DII score were used to categorize the subjects. The Chi-square test for qualitative variables and analysis of variance (ANOVA) for quantitative variables were applied to compare general features of study subjects across tertiles of DII score. Participants' energy-adjusted food intakes were compared using ANOVA across tertiles of DII scores. Energy adjustment was done using the residual approach that was explained previously by Willett et al.^39^. For calculating energy-adjusted dietary intakes, each of the dietary components is regressed on their total energy intake and residual values were added to their actual mean intake to estimate energy-adjusted values. Multiple linear regression analyses were applied to analyze the relationship between DII and headache frequency, duration, and serum NO levels, and the beta (β) estimate with the associated 95% confidence intervals (CIs) were reported. The relationship between DII and migraine severity and HIT-6 was assessed using multivariable logistic regression, and the odds ratios (OR) with corresponding 95% CIs were provided. Different confounders were controlled in this study. Model 1 was adjusted for age (continuous) and sex; then, marital status (single/married), smoking status (current smoker/non-current smoker), migraine characteristic (with aura/without aura), family history (yes/no), mean arterial pressure (continuous), and physical activity (continuous) were added. Finally, BMI (continuous) was added to Model 2, resulting in the creation of the third model. SPSS version 26 (SPSS Inc., Chicago, IL, USA, www.ibm.com) was used for all analyses (IBM Corp, Armonk, NY, USA). P values < 0.05 were considered statistically significant. The sample ample size was estimated on the basis of similar studies and suggested formula for a cross-sectional design using α = 0.05, β = 0.95, r = 0.25, and drop-out rate of 10% that yielded 265 subjects^29^.

### Ethics approval and consent to participate

The research ethics committee of Isfahan University of Medical Sciences approved the protocol of the current study on 26 August 2019 (IR.MUI.RESEARCH.REC.1398.352).

## Results

A total of 770 patients were evaluated and finally, 265 patients met our inclusion criteria and consented to be enrolled in this study. The further exclusion was made for 3 subjects, since their total energy intake, on the basis of FFQ, was outside the range of 800–4200 kcal/day. The flow chart of the participants’ selection process was shown in Fig. 1. The mean (SE) of BMI, age, and DII of the study population were 25.55 (0.21) kg/m^2^, 36.1 (0.53) years, and − 2.96 (0.06), respectively. The general features of the study population across tertiles of DII score are given in Table 1. Compared to individuals with the lowest DII scores, those with the highest values had augmented severity of headaches, increased headache frequency, and a greater probability of migraines without aura. No other significant differences were observed for studied parameters across tertiles of DII (all P values < 0.05).

**Table 1:** Characteristics of study population stratified by tertiles of dietary inflammatory index.

Variables	Tertiles of dietary inflammatory index			
	T1 [< − 3.52]	T2 [− 3.52 to – 2.47]	T3 [> − 2.47]	P value
N	87	88	87	
DII	− 4.04 ± 0.04	− 3.01 ± 0.03	− 1.83 ± 0.06	< 0.001
**Demographic variables**				
Age (y)	37.08 ± 0.81	35.77 ± 0.98	35.45 ± 0.96	0.423
Female	78 (89.7)	77 (87.5)	69 (79.3)	0.053
Married	75 (86.2)	68 (77.3)	69 (79.3)	0.248
Current smoker	4 (4.6)	3 (3.4)	8 (9.2)	0.193
Number of family members	3.40 ± 0.10	3.44 ± 0.10	3.40 ± 0.10	0.952
Weight (kg)	69.12 ± 1.12	66.01 ± 1.07	68.34 ± 1.23	0.136
Height (cm)	163.46 ± 0.83	161.25 ± 0.72	163.86 ± 0.92	0.057
BMI (kg/m^2^)	25.82 ± 0.34	25.43 ± 0.40	25.39 ± 0.35	0.665
Physical activity (MET/h/day)	9.20 ± 1.72	9.88 ± 2.88	7.54 ± 1.85	0.745
SBP (mmHg)	112.60 ± 1.09	113.40 ± 0.84	111.95 ± 1.16	0.614
DBP (mmHg)	75.24 ± 0.79	75.76 ± 0.70	75.44 ± 0.83	0.893
MAP (mmHg)	87.69 ± 0.83	88.31 ± 0.66	87.61 ± 0.86	0.794
**Migraine-related information**				
Migraine in first degree relatives	58 (66.7)	61 (69.3)	48 (55.2)	0.116
Time since migraine diagnosis (year)	7.96 ± 0.88	7.38 ± 0.98	6.65 ± 0.91	0.612
Migraine with aura	45 (51.7)	36 (40.9)	28 (32.2)	0.009
Frequency (attacks per month)	6.43 ± 0.46	7.22 ± 0.72	9.75 ± 0.94	0.004
Duration (day/attack)	1.07 ± 0.08	0.96 ± 0.09	0.85 ± 0.08	0.213
Subjects with severe headache	26 (29.9)	42 (47.7)	46 (52.9)	0.004
HIT-6 (score)	62.54 ± 0.76	62.93 ± 0.78	62.69 ± 0.75	0.936
Nitric oxide (nmol/mL)	35.50 ± 2.26	33.85 ± 2.22	33.04 ± 2.36	0.740
**Medications**				
Taking beta-blockers	36 (41.4)	29 (33.0)	43 (49.4)	0.282
Taking topitamate	3 (3.4)	8 (9.1)	2 (2.3)	0.728
Taking TCAs	44 (50.6)	38 (43.2)	40 (46.0)	0.544
Taking TeCAs	2 (2.3)	2 (2.3)	4 (4.6)	0.379
Taking SNRIs	3 (3.4)	4 (4.5)	7 (8.0)	0.178
Taking sodium valproate	8 (9.2)	10 (11.4)	15 (17.2)	0.110
Taking triptans	16 (18.4)	12 (13.6)	15 (17.2)	0.838
Taking gabapentin	15 (17.2)	13 (14.8)	15 (17.2)	> 0.99
Taking benzodiazepine	3 (3.4)	4 (4.5)	6 (6.9)	0.296

Data are presented as mean ± standard error or number (% within tertiles of dietary inflammatory index).

P value obtained from chi-square analysis for categorical variables and analysis of variance (ANOVA) for continuous variables.

*BMI* body mass index, *SBP* systolic blood pressure, *DBP* diastolic blood pressure, *MAP* mean arterial pressure, *TCA* tricyclic antidepressants, *TeCA* tetracyclic antidepressant, *SNRI* serotonin-norepinephrine reuptake inhibitor, *HIT* headache impact test, *DII* Dietary Inflammatory Index.

The energy-adjusted dietary intakes of participants across tertiles of DII are presented in Table 2. Compared to the lowest DII values, individuals with the highest DII scores consumed significantly higher amounts of energy, solid oils, fat, and sweets, as well as lower amounts of protein, carbohydrate, total fiber, potassium, magnesium, riboflavin, fruits, vegetables, nuts and seeds, fish, and legumes (all P values < 0.05).

**Table 2:** Selected food groups and nutrients intake of participants across tertiles of dietary inflammatory index*.

Variables	Tertiles of dietary inflammatory index			
	T1	T2	T3	P value
**Nutrients**				
Energy (Kcal/day)**	2496.86 ± 75.35	2600.36 ± 63.66	2857.32 ± 71.11	0.001
Protein (g/day)	78.96 ± 1.77	75.03 ± 1.79	65.79 ± 2.15	< 0.001
Fat (g/day)	102.63 ± 1.82	112.28 ± 1.91	117.47 ± 2.60	< 0.001
Carbohydrate (g/day)	379.04 ± 4.31	356.69 ± 4.30	349.26 ± 6.02	< 0.001
Total fiber (g/day)	24.54 ± 0.60	19.71 ± 0.43	15.30 ± 0.52	< 0.001
Potassium (mg/day)	4307.71 ± 92.13	3752.99 ± 66.00	2975.98 ± 86.89	< 0.001
Sodium (mg/day)	7593.09 ± 163.64	7691.31 ± 223.20	7273.76 ± 231.55	0.338
Magnesium (mg/day)	318.26 ± 5.14	284.20 ± 4.63	233.44 ± 5.38	< 0.001
Riboflavin (mg/day)	1.67 ± 0.06	1.67 ± 0.06	1.46 ± 0.06	0.046
**Food groups (g/day)**				
Fruits	671.54 ± 33.16	535.19 ± 20.61	411.46 ± 25.27	< 0.001
Vegetables	425.29 ± 26.43	306.57 ± 14.19	204.48 ± 13.40	< 0.001
Fish	5.08 ± 0.60	5.34 ± 0.67	2.61 ± 0.58	0.004
Whole grains	46.80 ± 5.44	40.26 ± 4.08	33.10 ± 4.98	0.143
Legumes	56.51 ± 5.85	40.25 ± 4.00	34.98 ± 4.16	0.004
Nuts and seeds	12.45 ± 1.40	9.83 ± 1.19	6.81 ± 1.14	0.008
Egg	25.66 ± 2.41	24.81 ± 2.33	18.57 ± 2.27	0.069
Solid oils	11.18 ± 1.39	18.64 ± 2.40	32.48 ± 3.61	< 0.001
Sweets	48.06 ± 3.61	52.02 ± 3.54	72.32 ± 6.51	0.001

Data are presented as mean ± standard error and obtained from analysis of variance (ANOVA).

P < 0.05 was considered statistically significant.

*All values have been adjusted for total energy intake using a residual method.

**Energy intake was not adjusted.

The β estimates and 95% CIs for the association between DII and serum NO, headache frequency, and duration are shown in Table 3. The crude model showed that individuals with a pro-inflammatory diet (as evidenced by higher values of DII) had increased headache frequency (β = 3.32; 95% CI 1.28, 5.35) compared to those with an anti-inflammatory diet. This association remained significant after controlling for age, sex, smoking status, marital status, family history, migraine characteristics, MAP, and physical activity (β = 3.49; 95% CI 1.44, 5.55). After further adjustment for BMI, an increase of 3.48 in headache frequency was observed when the DII score increased from − 4.04 to − 1.83 (β = 3.48; 95% CI 1.43, 5.54). Headache duration tended to be inversely associated with DII when comparing subjects in the third tertile of DII to those in the first tertile (β = − 0.22; 95% CI − 0.46, 0.02). However, controlling for potential confounders attenuated the findings (β = − 0.20; 95% CI − 0.44, 0.04). Moreover, no significant association was detected between DII and serum NO levels either before or after adjustment for confounders.

**Table 3:** Beta (β) and 95% confidence interval for serum nitric oxide, headache frequency, and duration across tertiles of the dietary inflammatory index.

	Tertiles of dietary inflammatory index			
	T1	T2	T3	P trend
**Frequency**				
Crude	Ref	0.79 (− 1.23, 2.81)	3.32 (1.28, 5.35)	0.001
Model 1	Ref	0.70 (− 1.32, 2.72)	3.16 (1.11, 5.20)	0.003
Model 2	Ref	0.89 (− 1.11, 2.90)	3.49 (1.44, 5.55)	0.001
Model 3	Ref	0.88 (− 1.12, 2.89)	3.48 (1.43, 5.54)	0.001
**Duration**				
Crude	Ref	− 0.11 (− 0.36, 0.12)	− 0.22 (− 0.46, 0.02)	0.076
Model 1	Ref	− 0.10 (− 0.34, 0.13)	− 0.17 (− 0.42, 0.06)	0.154
Model 2	Ref	− 0.11 (− 0.35, 0.12)	− 0.20 (− 0.44, 0.04)	0.106
Model 3	Ref	− 0.12 (− 0.35, 0.11)	− 0.20 (− 0.44, 0.04)	0.104
**Nitric oxide**				
Crude	Ref	− 1.65 (− 7.94, 4.63)	− 2.46 (− 8.77, 3.84)	0.444
Model 1	Ref	− 1.47 (− 7.71, 4.76)	− 2.74 (− 9.04, 3.56)	0.394
Model 2	Ref	− 1.85 (− 8.12, 4.40)	− 3.09 (− 9.49, 3.30)	0.343
Model 3	Ref	− 1.90 (− 8.15, 4.35)	− 3.13 (− 9.52, 3.25)	0.336

Data are presented as β (95% confidence interval) and obtained from linear regression.

Crude: Unadjusted.

Model 1: Adjusted for age and sex.

Model 2: Model 1 + marital status, smoking status, migraine characteristic, family history, mean arterial pressure, and physical activity.

Model 3: Model 2 + body mass index.

*HIT* Headache Impact Test.

P < 0.05 was considered statistically significant.

In Table 4, the OR and 95% CIs for headache severity and HIT-6 across tertiles of the DII are indicated. The crude model revealed that individuals with the highest adherence to a pro-inflammatory diet had a higher likelihood of severe headache (OR = 2.53; 95% CI 1.36, 4.71) compared to those with the lowest adherence. Furthermore, after adjusting for age and sex, participants with the greatest DII score had higher odds of severe headache (OR = 2.32; 95% CI 1.23, 4.36) than those with the lowest DII values. Additional adjustments for marital status, migraine characteristics, family history, smoking status, MAP, and physical activity revealed that individuals in the third tertile of DII had a higher risk of severe headaches (OR = 2.25; 95% CI 1.17, 4.32) than those in the first tertile. Finally, when we added BMI as a confounder, a significant association was observed similar to the previous model (OR = 2.25; 95% CI 1.17, 4.32). No significant association was observed in terms of DII and HIT-6.

**Table 4:** Odds ratio (OR) and 95% confidence interval for headache severity and HIT-6 across tertiles of the dietary inflammatory index.

	Tertiles of dietary inflammatory index			
	T1	T2	T3	P trend
**Severity**				
Crude	Ref	2.11 (1.14, 3.91)	2.53 (1.36, 4.71)	0.003
Model 1	Ref	2.04 (1.09, 3.80)	2.32 (1.23, 4.36)	0.009
Model 2	Ref	1.92 (1.01, 3.63)	2.25 (1.17, 4.32)	0.014
Model 3	Ref	1.91 (1.01, 3.64)	2.25 (1.17, 4.32)	0.015
**HIT-6**				
Crude	Ref	1.002 (0.53, 1.88)	0.90 (0.48, 1.68)	0.748
Model 1	Ref	1.006 (0.53, 1.89)	0.93 (0.49, 1.76)	0.843
Model 2	Ref	1.06 (0.55, 2.04)	1.03 (0.53, 1.98)	0.929
Model 3	Ref	1.04 (0.54, 1.99)	1.02 (0.53, 1.96)	0.949

Data are presented as odds ratio (95% confidence interval) and obtained from logistic regression.

Crude: Unadjusted.

Model 1: Adjusted for age and sex.

Model 2: Model 1 + marital status, smoking status, migraine characteristic, family history, mean arterial pressure, and physical activity.

Model 3: Model 2 + body mass index.

*HIT* Headache Impact Test.

P < 0.05 was considered statistically significant.

## Discussion

The current study showed a significant positive relationship between DII score and headache frequency. However, the results did not present a significant relationship between headache duration or nitric oxide and DII after adjusting for confounders. Also, diets with higher pro-inflammatory properties, which are associated with higher serum inflammatory markers, were significantly correlated with an increased risk of severe migraine headaches. However, no significant relationship was seen between DII and HIT-6. To the best of our knowledge, this study was one of the first to investigate the association between DII and migraine.

In the current study, various possible confounders were selected to be adjusted in the regression models including age, sex, marital status, smoking status, migraine characteristic, family history, mean arterial pressure, physical activity, and BMI. The logic for using these variables was mostly based on similar studies and also literature search^40–42^. Moreover, due to the substantial role of adiposity in migraine^43,44^, BMI was added in a separate model to detect an adiposity independent association.

Previous research has indicated that some food patterns consistent with lower DII scores are associated with fewer migraine headache-related parameters. For example, the Dietary Approaches to Stop Hypertension (DASH) diet, which has low DII, was previously correlated with reduced headache severity and duration among migraine patients^45^. The majority of studies investigating the effects of nutrition on migraines have focused on the association between different types of foods and nutrients. Studies suggest that consumption of anti-inflammatory food items, including seafood^46^, ginger^47^, pepper, garlic, and onions^48^, is associated with improved clinical findings in patients suffering from migraine headaches. Moreover, the DII has previously been associated with other neurological disorders including dementia^49^, memory function^50^, and cognitive function^51^. Few studies have directly evaluated the association between migraine parameters and DII scores. One study considering the effects of the DII score on headaches among 266 Iranian women aged 18–45 years (DII score range − 4.22 to + 5.19) indicated a direct association between the DII score and headache frequency after adjustment for potential confounders. However, no relationship was found between headache duration or migraine severity and DII score^45^. Furthermore, a few studies have demonstrated increased inflammatory markers throughout the episodes of a migraine attack^52–54^. However, other studies have found no association between inflammatory markers and migraine headache severity or duration^55^. The discrepancy in results may be due to differences in study design, sample size, geographic area, and method of calculating the DII.

Migraines are described as a headache disorder with a significant effect on the quality of life and are prevalent in both developing and developed countries^56^. The mechanisms underlying migraine headaches are unresolved but are hypothesized to involve a degree of inflammation. The DII score is an index that reflects potential dietary inflammatory properties based on six inflammatory biomarkers, including Interleukins (IL)-1β, IL-6, IL-4, IL-10; Tumor Necrosis Factor (TNF)-α; and C-Reactive Protein (CRP)^57^. Secretion of Calcitonin Gene-Related Peptide (CGRP) leads to enhancement in the activity of NO synthase induction (iNOs), generation of NO expression, the activity of the cyclooxygenase-2 (COX-2) enzyme, and cytokine secretion of inflammatory factors such as IL-6, TNF-α, and IL-1β^58^. It has been established that cytokines, through enhanced permeability and cell-to-cell interaction, have been shown to play a major role in the pathogenesis of inflammation and pain associated with migraines^59^. In patients experiencing migraines, vascular disorder leads to endothelial activation and increased generation of factors such as inflammatory cytokines, intercellular adhesion molecule (ICAM), and vascular cell adhesion molecule-1 (VCAM). Pro-inflammatory cytokines, such as TNF-α, are vasodilators and induce the expression of ICAM and VCAM. Greater expression of these factors is related to microglia, which activates neuropathic inflammation and pain in the brain^60–62^. Also, some studies have indicated an association between consumption of magnesium^63^, riboflavin^64^ fruits, and vegetables^65^ and migraine. Our results showed that individuals in the lowest tertile of DII consumed significantly higher amounts of magnesium, riboflavin, fruits, and vegetables. As a result, higher consumption of these items may be a potential reason to explain migraine-related improvements in the present study.

We implemented a rigorous methodology in terms of patients’ selection, diagnosis of migraine, and use of valid and reliable questionnaires to improve the internal validity of the current study. Additionally, lots of possible confounders were controlled to show that the association was independent of confounders. However, due to residual confounders and some known questionnaire-based bias, this issue should be taken into account while interpreting the internal validity. In terms of external validity (generalizability), the findings of the current study can be generalized to the Iranian population with migraine headache which is independent of their sex, age, BMI, marital status, smoking, migraine characteristic, family history, MAP, and physical activity. However, the result must be generalized with caution to other populations.

The present study has several strengths. Various potential confounders were controlled for in this analysis, leading to unbiased risk estimates in this study. A validated FFQ was applied for the evaluation of dietary intakes. Nevertheless, limitations are also present. Notably, the cross-sectional design does not allow for the exploration of causative relationships between the DII and migraine. The FFQ is a memory-based questionnaire that is approximate in nature; therefore, another limitation is the potential overestimation or underestimation of dietary intake. Furthermore, residual confounders cannot be excluded, even when the model is adjusted for multiple probable confounders. While the socio-economic status of the study population was representative of the general Iranian population, generalizing these results to other populations should be done cautiously. In another word, the current study population may differ from others in terms of specific habits, culture, tradition, and geographical location that can influence our findings and diminish the generalizability of our results to other study populations. Moreover, patients were asked to refer to the laboratory for blood sampling on a headache-free day; however, it is possible that all of them were not headache free that this issue can influence our findings.

## Conclusion

In conclusion, we found evidence showing that higher adherence to a diet with anti-inflammatory properties was significantly and inversely related to headache frequency. Furthermore, our results suggest that the DII score is significantly associated with the severity of migraines. More studies with larger sample sizes are needed to confirm these findings.

## Data Availability

The data that support the findings of this study are available from the corresponding author upon reasonable request.
